# *Icriodus marieae*, a new icriodontid conodont species from the Middle Devonian

**DOI:** 10.1007/s12542-017-0337-9

**Published:** 2017-02-21

**Authors:** Thomas J. Suttner, Erika Kido, Andreas W. W. Suttner

**Affiliations:** 1 Institute of Earth Sciences, NAWI-Graz, University of Graz, Heinrichstraße 26, 8010 Graz, Austria; 2 Geological-Paleontological Department, Natural History Museum Vienna, Burgring 7, 1010 Vienna, Austria; 3 Kollergasse, 1030 Vienna, Austria

**Keywords:** Conodonta, Prioniodontida, Icriodontidae, Middle Devonian, Carnic Alps, Conodonta, Prioniodontida, Icriodontidae, Mittel-Devon, Karnische Alpen

## Abstract

A new conodont species, *Icriodus marieae*, is described from pelagic limestone beds of the Carnic Alps (Austria). Specimens are obtained from the upper part of the Valentin Formation (Central Carnic Alps) and range from the latest Eifelian to middle Givetian. Significantly differing from other icriodontid conodonts is that the icriodontan element of the new species develops only three denticles on either lateral denticle row, which are constricted to the central part of the element. The anterior part of the element is free of lateral row denticles and consists of two to four denticles, which have a fan-shaped outline in lateral view. The anterior part as well as the posterior part (consisting of cusp and two to three pre-cusp denticles) is higher than the denticles of the central part of the element. Shape analysis confirms that the parameters chosen for landmarks (element size relation and denticle setting) show little variation between different specimens.

## Kurzfassung

Mit *Icriodus marieae* wird eine neue Conodonten-Art aus pelagischen Kalken der Karnischen Alpen (Österreich) beschrieben. Conodonten-Elemente dieser Art stammen aus dem oberen Anteil der Valentin-Formation (Zentralkarnische Alpen) und reichen vom späten Eifelium bis in das mittlere Givetium. Im Gegensatz zu anderen icriodontiden Arten hat das I-Element der neu aufgestellten Art nur drei Dentikel links und rechts der medianen Reihe ausgebildet, welche in Oralansicht eine zentrale Position einnehmen. Der vordere Anteil des Elementes besteht aus zwei bis vier Dentikel der medianen Reihe, die in Seitenansicht eine fächerförmige Silhouette ergeben. Der vordere sowie auch der hintere Anteil (letzterer setzt sich aus Hauptzahn und zwei bis drei, dem Hauptzahn vorangestellten, Knoten zusammen) sind höher als der mit Lateral-Leisten-Dentikel besetzte zentrale Anteil des Elementes. Eine Größen-korrelierte Analyse zeigt, dass Abstand und Position der Dentikel in den gemessenen Elementen nur gering variiert.

## Introduction

Restudy of a condensed pelagic section for a higher-resolution stratigraphy around the Eifelian–Givetian boundary of the Valentin Formation in the Carnic Alps (Schönlaub et al. 1980; Göddertz 1982; Hüneke 2007; Spalletta et al. 2015) resulted in a diverse conodont fauna. Currently, our work is based on existing conodont distribution charts subsequently updated by Schönlaub and colleagues (see: Schönlaub et al. 1980, 1997, 2004; Schönlaub 1999; Göddertz 1982). Different from earlier studies, we split and dissolved single layers of each bed (sampled interval: from bed 69 to bed 73, with the E/G boundary tentatively set between beds 70/71 and the G/F boundary set between beds 72/73 by Schönlaub et al. 1980). Bedding planes are recognised as thin stylolite layers (iron–manganese crusts which indicate time intervals of reduced sedimentation).

During conodont identification, we noticed that some of the icriodontid specimens distinctively have lateral row denticles constricted to the central part of the icriodontan element with the anterior part forming a fan-shaped outline in lateral view. One such specimen was already illustrated by Schönlaub et al. (1980, pl. 9, figs. 11, 12) and identified as *Icriodus brevis*. Weddige (1990) described the phenomenon of reduced development of lateral row denticles in his article on pathological conodonts as *Occlusio*. According to his hypothesis, it represents an ontogenetically gained asymmetric deformation as a result of antagonistic activity of element pairs. Because we found 22 specimens (and one more illustrated by Schönlaub et al. 1980) including left and right elements of different size, which all show the same features but lack distinct tip wear or other occlusal deformation in the anterior part, we think that the reduction of lateral row denticles reflects a phylogenetic rather than ontogenetic deformation.

## Geological settings

### Locality

**Fig. 1:**
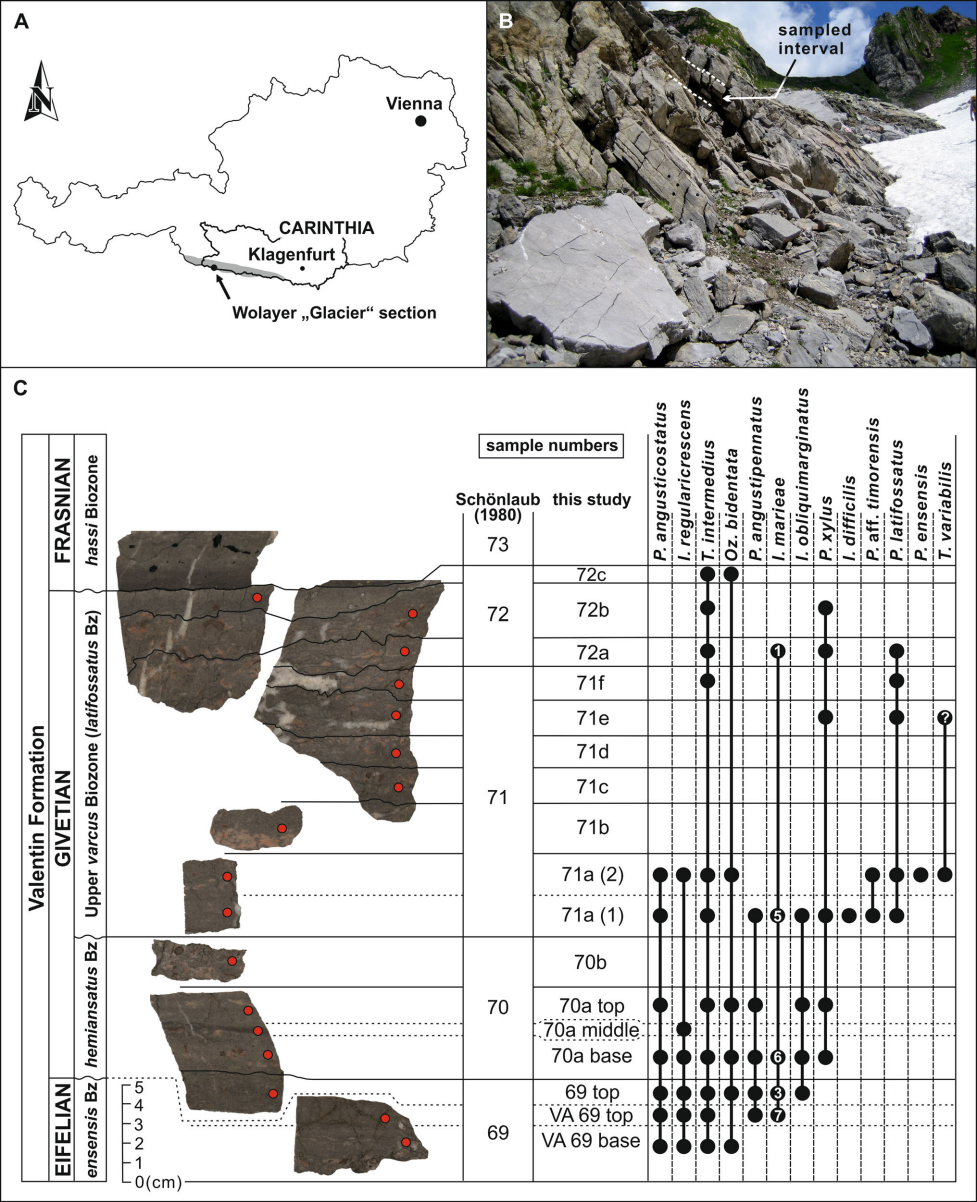
Type locality and stratigraphic range of *Icriodus marieae* sp. nov. **a** Simplified map of Austria with the Wolayer “Glacier” section (Wolayer Valley, Carnic Alps) indicated. The area of the Carnic Alps is highlighted in *grey*. **b** Photo of the Wolayer “Glacier” section (=Valentin Formation type section). The sampled interval is located at the top of the formation (*arrowhead*). **c** Polished slabs of the sampled interval with bed/layer numbers and the range of stratigraphically most important conodont taxa indicated. *Bz* biozone, *P*. Polygnathus, *I.* Icriodus, *T*. Tortodus, *Oz.* Ozarkodina

The studied material derived from the Wolayer “Glacier” section (Fig. 1a, b; N 46°36′49.0″, E 12°52′34.7″), which is the nominated type section of the Valentin Formation (Spalletta et al. 2015). It is located along the northern side of the Wolayer Valley in high alpine terrain of the Carnic Alps (Carinthia, Austria) and can easily be reached either via the path to Lake Wolayer from the Lower Valentinalm or from Lake Wolayer along the path to the Valentintörl. All samples were taken from near the top of the formation (indicated by arrowhead in Fig. 1b).

### Stratigraphy and lithology

The Valentin Formation is deposited above the Findenig Formation and succeeded by the Pal Grande Formation (Fig. 1b). It consists of thin bedded greyish bioclastic wackestone and packstone with a marking phosphorite horizon at its top. The unit represents a strongly condensed pelagic depositional environment that reaches about 15 meters in thickness at its type section. Based on conodont biostratigraphy, an age from Emsian (*serotinus* Biozone) to Frasnian (Lower *hassi* Biozone) is suggested (Schönlaub et al. 1980; updated by Hüneke 2007). However, the Eifelian–Givetian boundary is located close to the top of the formation. Based on ultra-high-resolution sampling, we aimed to determine a more detailed biostratigraphy between beds 69 and 73, with the E/G boundary allocated between samples VA 69 top and 69 top (Fig. 1c). The boundary is set based on the first occurrence of *Icriodus obliquimarginatus* in sample 69 top. The Givetian–Frasnian boundary is located between beds 72 and 73 (Schönlaub et al. 1980). Further characterisation of the Valentin Formation is provided by Spalletta et al. (2015, and references therein).

It should be noted that several beds yield mixed conodont faunas which due to reworking of slightly older sediments have been redeposited in the Valentin Formation. However, in Fig. 1, we provide occurrence raw data of conodonts extracted from the limestones of the Valentin Formation. Subsequent study related to conodont biodiversity and microfacies shall clarify the real range (especially the last occurrence datum) of *Icriodus marieae*, which seems to be biased by reworking in beds 71 and 72.

## Materials and methods

Conodonts were extracted from 21 limestone samples in the sieving laboratory of the Institute of Earth Sciences (University of Graz, Austria) following standard chemical extraction methods for phosphatic microfossils (e.g. Jeppsson and Anehus 1995). Rock material was dissolved in sieves placed in 5-L buckets under a concentration of about 5 percent HCOOH (technical grade: 85 percent) diluted in 95 percent of well-temperate tap water (38–40 °C) until the chemical reaction stopped. Because tap water used for extraction procedures contains high concentrations of calcium and magnesium (15°–17°dH), no calcium carbonate buffer was added. The procedure was repeated two times a day and lasted about 2–4 days depending on the sample size (weight of samples ranged from below 50 to 400 g). Because the amount of residue per sample was small after wet sieving (separated into three fractions: 63, 125 + 250 and 500 µm), no heavy liquid treatment was applied. After drying insoluble residues in a drying oven at 50 °C, picking and determination of conodont elements was done under an Olympus SZ40 stereomicroscope. For better recognition of micro-ornamentation and illustration of specimens, scanning electron microscopy (SEM) images were obtained using a DSM 982 Gemini (Zeiss).

**Fig. 2:**
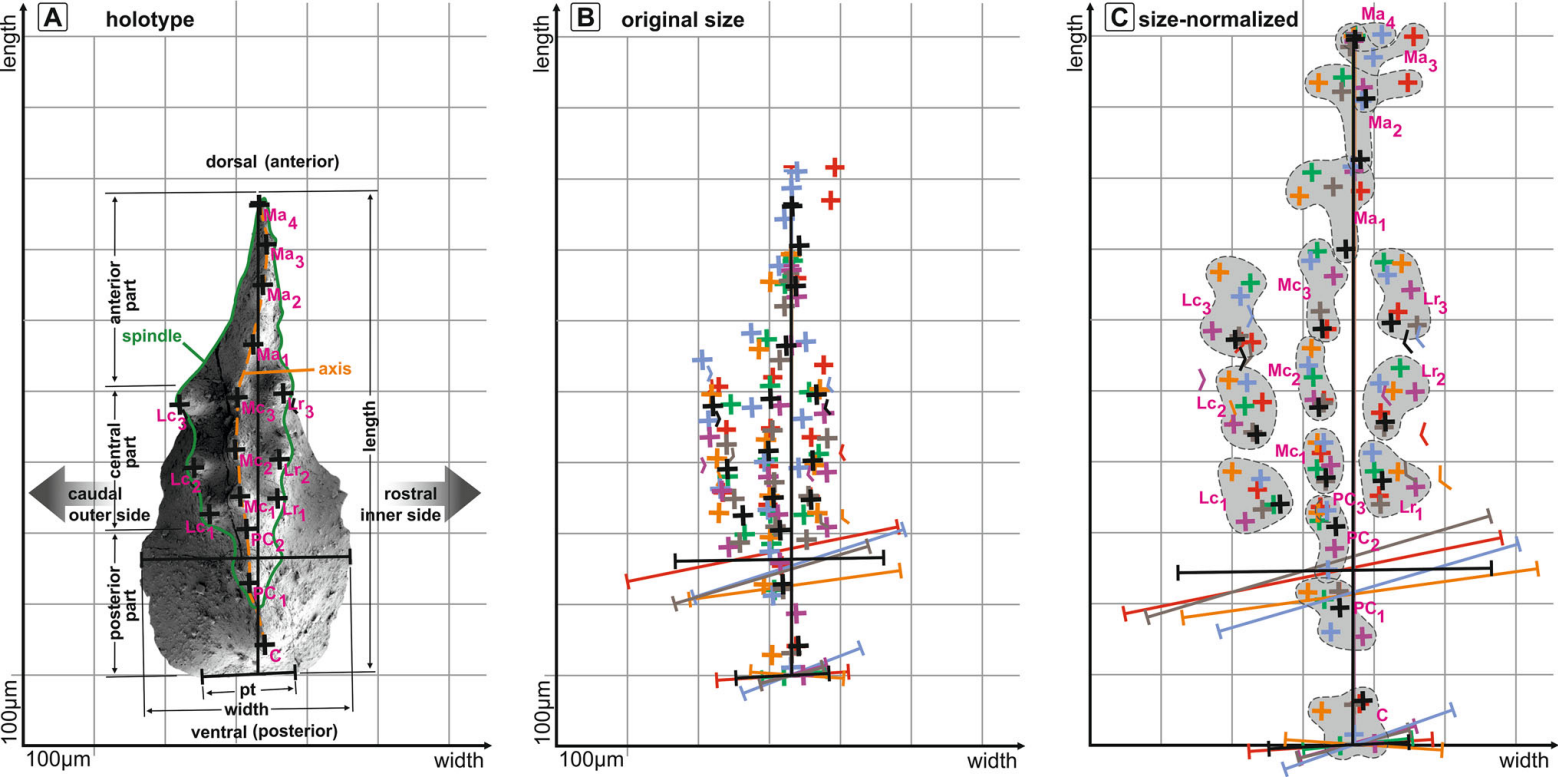
*Icriodus marieae* sp. nov., shape analysis. **a** Oral view of the holotype including landmarks and orientation; **b**, landmarks and measurement of length, width, and width of posterior termination in original size of each element; **c**, for better comparison, I elements are rescaled and normalised to elements length = 100 %. *C*  cusp, *pC*_*1–3*_ pre-cusp 1–3, *Lc*_*1–3*_ lateral row caudal denticle 1–3, *Lr*_*1–3*_ lateral row rostral denticle 1–3, *Mc*_*1–3*_ median row central denticle 1–3, *Ma*_*1–4*_ median row anterior denticle 1–4, *pt* posterior termination

For documentation of intraspecific variation of the newly established species, we applied shape analysis of the oral surface of most of the specimens using tips of lateral row, median row, cusp and pre-cusp denticles, elements length, maximum width, and width of the posterior termination as parameters (Fig. 2).

### Repository

All specimens (holotype and paratypes) are stored at the Geological Survey of Austria in Vienna (room 1-41-00; repository numbers: GBA-2016/013/0001/001 to GBA-2016/013/0007/050).

## Systematic palaeontology

This published work and the nomenclatural acts it contains have been registered with Zoobank: http://zoobank.org/References/7D4349C7-B6E9-4E3F-A5A8-3775EBCDCEC7.

Phylum **Chordata** Bateson, 1886.

Class **Conodonta** Eichenberg, 1930 sensu Sweet and Donoghue, 2001.

Order **Prioniodontida** Dzik, 1976.

Family **Icriodontidae** Müller and Müller, 1957.

Genus ***Icriodus*** Branson and Mehl, 1938.

*Type species.* *Icriodus expansus* Branson and Mehl, 1938; Givetian to Frasnian; from the Mineola Limestone of Missouri, United States of America.

*Description.* Apparatus consists of one pair of I (icriodontan) and an uncertain amount of S (acodinan) elements. The spindle of icriodontan elements characteristically consists of three longitudinal rows of denticles which can be connected through transverse ridges and are followed by one or more prominent denticles in posterior extension of the median row (cusp; pre-cusp). The basal cavity is opened widely either with a flaring rim near the lower margin, or being straight. S elements are coniform (geniculate or non-geniculate) and possess a smooth or ornamented (e.g. striate) surface. Important remarks and further readings regarding the apparatus composition of this genus are provided by Bultynck (2003, pp. 304–306).

*Stratigraphic and geographic distribution.* Early Devonian (Emsian) to Late Devonian (Famennian) of Europe, Asia, Africa, Australia and North America.

***Icriodus marieae*** sp. nov.

Figure 3a–r.

**Fig. 3:**
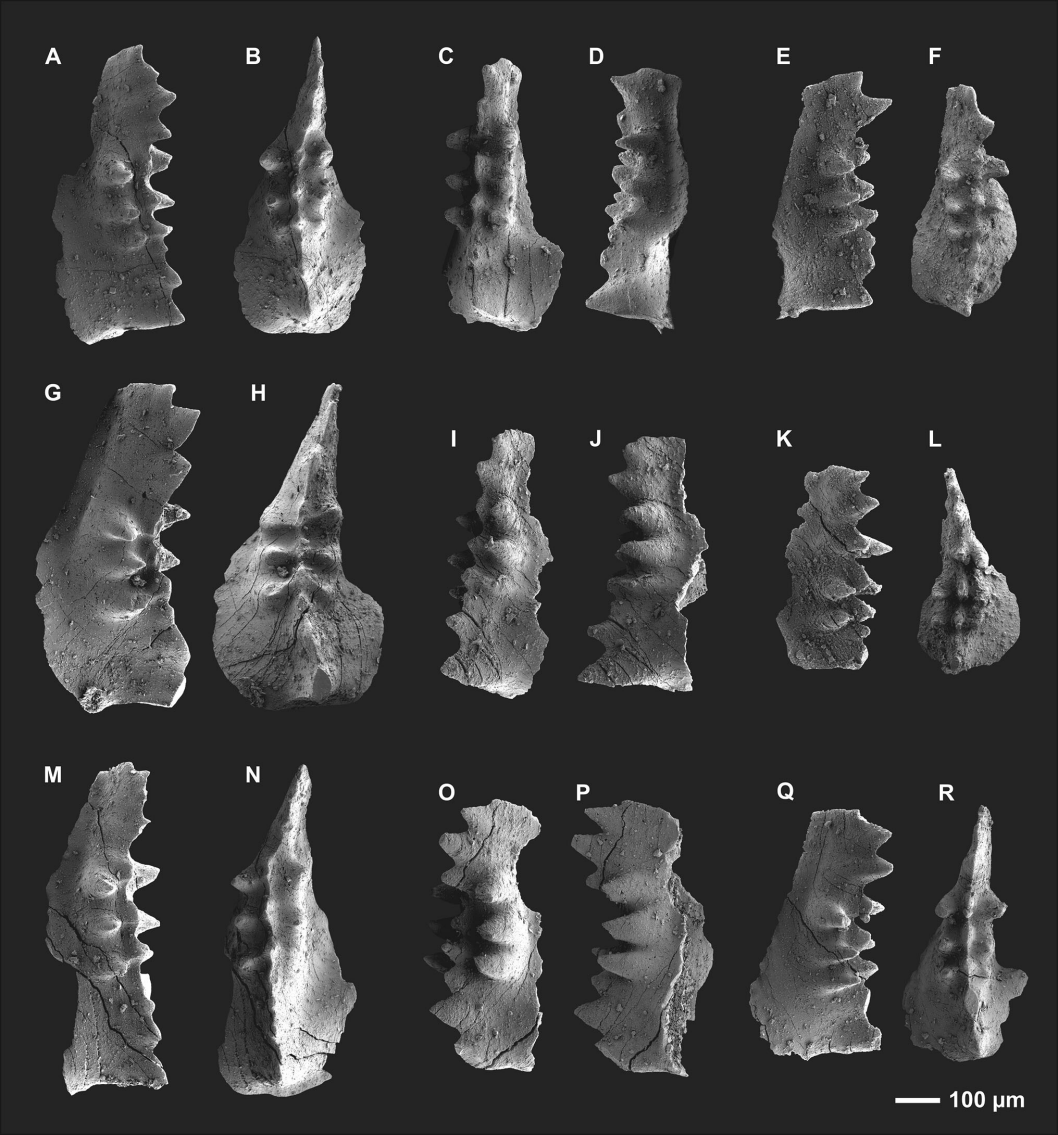
*Icriodus marieae* sp. nov., Valentin Formation, Eifelian–Givetian, Wolayer “Glacier” section, Carnic Alps, Austria. **a**, **b** Left I element, lateral and oral view, sample VA 69 top, B (holotype; GBA-2016/013/0003/010); **c**, **d** left I element, oral and lateral view, sample 69 top, O (paratype; GBA-2016/013/0004/018); **e**, **f** right I element, lateral and oral view, sample VA 69 top (paratype; GBA-2016/013/0003/013); **g**, **h** left I element, lateral and oral view, sample VA 69 top, R (paratype; GBA-2016/013/0003/009); **i**, **j** left I element, oral and lateral view, sample 69 top, G (paratype; GBA-2016/013/0004/021); **k**, **l** right I element, lateral and oral view, sample 69 top (paratype; GBA-2016/013/0004/014); **m**, **n** left I element, lateral and oral view, sample 70a base, BL (paratype; GBA-2016/013/0004/035); **o**, **p** left I element, oblique and lateral view, sample 70a base, P (paratype; GBA-2016/013/0004/037); **q**, **r** left I element, lateral and oral view, sample 70a base, BR (paratype; GBA-2016/013/0004/038). Abbreviations: prefix VA of the sample number = Valentin Formation additional sample; capital letters after the sample number indicate the colour used for landmarks in Fig. 2 of corresponding left I elements figured here (*B* black, *BL* blue, *BR* brown, *G* green, *O* orange, *P* purple, *R* red)

aff. 1972 *Icriodus eslaensis* van Adrichem Boogaert—Bultynck: p. 81, fig. 14E.

1980 *Icriodus brevis* Stauffer—Schönlaub et al.: pl. 9, figs. 11–12.

1990 *Icriodus brevis* Stauffer—Weddige: p. 572, pl. 3, figs. 2, 7.

*LSID*. urn:lsid:zoobank.org:pub:7D4349C7-B6E9-4E3F-A5A8-3775EBCDCEC7.

*Etymology.* Dedicated to Marie Suttner (30.03.1957–18.10.2001), mother of Andreas W. W. and Thomas J. Suttner.

*Type material.* Holotype: GBA-2016/013/0003/010. Paratypes: GBA-2016/013/0003/009, GBA-2016/013/0003/013, GBA-2016/013/0004/014, GBA-2016/013/0004/018, GBA-2016/013/0004/021, GBA-2016/013/0004/035, GBA-2016/013/0004/037, GBA-2016/013/0004/038; not illustrated paratypes: GBA-2016/013/0001/001–004, GBA-2016/013/0002/001–003, GBA-2016/013/0003/001–005, GBA-2016/013/0007/050.

*Studied material.* 22 icriodontan elements.

*Locality and horizon.* Wolayer “Glacier” section, Wolayer Valley, Carnic Alps (Carinthia, Austria). Bed 69 (sample numbers: VA 69 top, 69 top), bed 70 (sample number: 70a base), bed 71 [sample number: 71a (1)] and bed 72 (sample number: 72a) of the Valentin Formation.

*Diagnosis.* Lateral row denticles of icriodontan elements are constricted to the central part of the element. Commonly, each lateral row consists of three denticles, which are more or less of equal height with transversal connecting median row denticles. Anteriormost median row denticles are higher than those of the central median row and have a fan-shaped/cockscomb-like outline in lateral view. The posterior part of specimens bears large cusp and two to three pre-cusp denticles which, similar to denticles of the anterior part, are higher than denticles of the central part.

*Description.* The outline of the basal cavity is asymmetric and has a weak spur developed on the inner side which is followed by a more or less pronounced sinus that joins the spindle between the first and third denticle of the lateral row (Lr_1–3_) in oral view (Fig. 3h). The basal cavity outline of the outer side is semicircular between the posterior termination and the first denticle of the lateral row (pt–Lc_1_) and gradually joins the spindle at the third denticle of the lateral row (Lc_1_–Lc_3_). The posterior termination is quite broad, measuring about one-third to half the length of the elements maximum width. The maximum width is located between the first and third pre-cusp (PC_1–3_). The relation between maximum width and length is approximately 1:2. The relation of posterior, central and anterior part along the arcuate and sometimes slightly sigmoidal axis is nearly of equal length (C-PC_3_:Mc_1–3_:Ma_1–4_ = 1:1.3:1), with the central part (Mc_1–3_) being slightly longer (Fig. 2a). The spindle shape is elongate oval, being widest either at central position (at Lc_2_–Mc_2_–Lr_2_) or slightly anterior mid-length (at Lc_3_–Mc_3_–Lr_3_).

Significant for this species is the development of 3 denticles on either lateral row, which are constricted to the central part of the element. Denticles are discrete and well rounded. Some specimens (including the holotype) possess slightly smaller posteriormost lateral row denticles (Lc_1_ and Lr_1_) compared with remaining denticles of either lateral row (Lc_2–3_ and Lr_2–3_). In few specimens, single lateral row denticles are less well pronounced or developed as rudimentary node only (Fig. 3e, f: Lc_1_, K–L: Lr_3_, M–N: Lr_1_). Lateral row denticles are connected with less well-developed median row denticles/nodes (circular to ovate shape in oral view) by weak to pronounced ridges. The development of ridges (also axial ridges between median row denticles) is variable and could be related to specimen’s ontogeny. The larger the elements, the more pronounced are the ridges (Fig. 3g, h versus q, r). Transversal denticle rows are not in line and possess slightly offset median row denticles covering a position slightly anterior to the lateral row denticles (e.g. Fig. 3h: Lc_1_–Mc_1_–Lr_1_). Anterior median row denticles are transversally adpressed and ovate in oral view and much larger than central median row denticles in lateral view. The elements outline in lateral view shows a slightly arcuate lower margin until the basal cavity becomes adpressed and joins the spindle (posterior half to two-thirds); from then on, the lower margin continues straight until the anterior termination (angle at the junction between posterior lower margin and anterior lower margin varies from 160° to 170°). Elements possess a large cusp which is slightly inclined posteriorward. Although the tip of the cusp is broken in some specimens, it is evident that the cusp is highest and followed by pre-cusp denticles subsequently decreasing in size anteriorward. However, that feature is variable, hence some of the specimens possess a posterior portion with the first pre-cusp denticle being either of equal height, or slightly higher than the cusp. Central median row (Mc_1–3_) and adjacent lateral row denticles (Lc_1–3_ and Lr_1–3_) are lowest and more or less of equal height. The anterior median row develops three to four denticles which are high again and form a fan-shaped/cockscomb-like outline in lateral view. At least two of these are well developed, and can be recognised easily. They are triangular in shape and followed by an initial anteriormost denticle, which often is represented by a weakly developed node (e.g. Fig. 3d–j). All denticles including the cusp are smooth and do not show any micro-ornamentation.

### Remarks

A very similar element is sketched by Bultynck (1972, Fig. 14E) from the *varcus* Biozone (zone Gi c) collected from Mont d’Haurs (compare: Bonte and Ricour 1948; Lecompte 1960). The only feature which is not observed in Bultynck’s specimen is the fan-shaped outline of anterior median row denticles. Originally, that and some other specimens figured by Bultynck (1972, fig. 14a, e–f) were identified as *Icriodus eslaensis*, but later synonymised with *Icriodus lindensis* by Weddige (1977). In the same publication, Weddige (1977, p. 293) commented that Klapper et al. (1975, p. 89–90) correctly synonymised *Icriodus eslaensis* with *Icriodus brevis*. The cusp of *I. brevis* is lower than the subsequently following pre-cusp and less inclined posteriorward, which discriminates *I. brevis* from *I. lindensis*. Compared with *I. lindensis*, the cusp of *I. marieae* is commonly higher than the pre-cusp and less inclined.

Schönlaub et al. (1980) figured a representative of the newly erected species as *Icriodus brevis* from bed 72 of the Wolayer “Glacier” section. Weddige (1990) concluded that such elements are the result of what he termed *Occlusio* pathology. Two icriodontan elements identified as *Icriodus brevis* and figured on pl. 3, Figs. 2 and 7 (Weddige 1990) are strikingly similar to *Icriodus marieae*. The element figured on pl. 3, Fig. 2 differs only in possessing a slightly wider “blade” between the anteriormost lateral row denticle (Lc_3_) and the mid-length of the anterior portion (between Ma_2_ and Ma_3_), and in developing five anterior median row denticles.

At first glance, the newly erected species looks similar to one of the paratypes of *Icriodus calvini* introduced by Klapper and Barrick (1983, pp. 1231–1234, Fig. 10i–j). However, *Icriodus marieae* differs from the latter by possessing a wider basal cavity with a less well-developed spur. The major difference is that *I. calvini* has an extremely prominent double cusp which together with the pre-cusp is strongly inclined posteriorward.

*Stratigraphic and geographic distribution.* Icriodontan elements of the species are found in the Middle Devonian of the Carnic Alps, Austria (possible maximum range: *kockelianus*?/*ensensis*-Upper *varcus* Biozones), the Ardennes, France (*varcus* Biozone) and the Rhenish Mountains, Germany (*varcus* Biozone).

## Discussion

Weddige (1990) already recognised that some Middle Devonian icriodontid I elements show an atypical habitus of reducing lateral row denticles in the anterior part of the element. Weddige (1990) concluded that such elements show pathological occlusion. If this were the result of ontogenetically gained deformation caused by antagonistic activity of element pairs, the surface of the denticle remnants would show specific wear. However, this is not observed in specimens from the Valentin Formation. Studied material completely lacks anterior lateral row denticles, with the anterior part of the element being smooth on either side. But it must be remarked that, in some specimens, single lateral row denticles are less well pronounced or developed as rudimentary node only, which led to the conclusion that the loss of anterior lateral row denticles probably expresses a variation in the genetic make-up of some icriodontid species. However, the amount of morphologically similar elements found in only a few beds of the same section is significantly high and suggests that the phenotypic variation observed in these elements demarcates another evolutionary step and is not related to a sporadically occurring phenomenon, which we think justifies the introduction of a new taxon.

## Abbreviation

GBA: Geologische Bundesanstalt in Wien
